# A Wrench in the Works of Human Acetylcholinesterase: Soman Induced Conformational Changes Revealed by Molecular Dynamics Simulations

**DOI:** 10.1371/journal.pone.0121092

**Published:** 2015-04-13

**Authors:** Brian J. Bennion, Sebnem G. Essiz, Edmond Y. Lau, Jean-Luc Fattebert, Aiyana Emigh, Felice C. Lightstone

**Affiliations:** 1 Biosciences and Biotechnology Division, Lawrence Livermore National Laboratory, 7000 East Ave, Livermore CA, United States of America; 2 Bioinformatics and Genetics Department, Faculty of Engineering and Natural Sciences, Kadir Has University, 34083 Fatih, Istanbul, Turkey; 3 Center for Applied Scientific Computing, Lawrence Livermore National Laboratory, 7000 East Ave, Livermore CA, United States of America; Wake Forest University, UNITED STATES

## Abstract

Irreversible inactivation of human acetylcholinesterase (hAChE) by organophosphorous pesticides (OPs) and chemical weapon agents (CWA) has severe morbidity and mortality consequences. We present data from quantum mechanics/molecular mechanics (QM/MM) and 80 classical molecular dynamics (MD) simulations of the apo and soman-adducted forms of hAChE to investigate the effects on the dynamics and protein structure when the catalytic Serine 203 is phosphonylated. We find that the soman phosphonylation of the active site Ser203 follows a water assisted addition-elimination mechanism with the elimination of the fluoride ion being the highest energy barrier at 6.5 kcal/mole. We observe soman-dependent changes in backbone and sidechain motions compared to the apo form of the protein. These alterations restrict the soman-adducted hAChE to a structural state that is primed for the soman adduct to be cleaved and removed from the active site. The altered motions and resulting structures provide alternative pathways into and out of the hAChE active site. In the soman-adducted protein both side and back door pathways are viable for soman adduct access. Correlation analysis of the apo and soman adducted MD trajectories shows that the correlation of gorge entrance and back door motion is disrupted when hAChE is adducted. This supports the hypothesis that substrate and product can use two different pathways as entry and exit sites in the apo form of the protein. These alternative pathways have important implications for the rational design of medical countermeasures.

## Introduction

**Fig 1:**
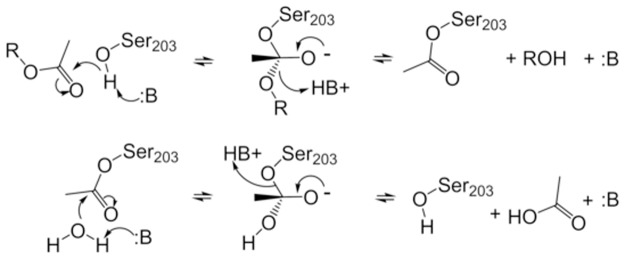
Acylation mechanism of hAChE by acetylcholine. The nucleophilic attack of the deprotonated Ser203 hydroxy group on the carbonyl carbon of acetylcholine and the reactivation of Ser203 by an activated water is shown.

**Fig 2:**
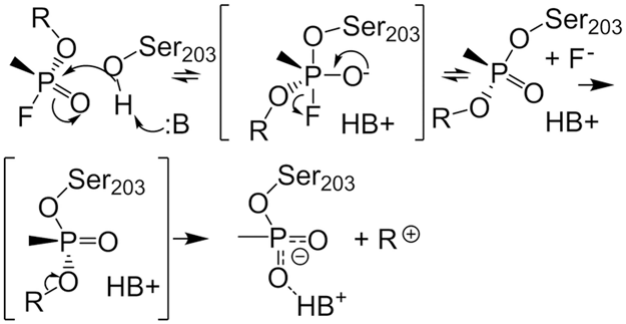
Mechanisms of soman phosphonylation and subsequent ageing of hAChE. The nucleophilic attack of the deprotonated Ser203 hydroxyl group on the phosphorous atom of soman. The leaving group is the fluoride ion. Ageing is shown as the cleavage of the pinacolyl R-group from the phosphorous atom.

The acetylcholinesterase enzyme (AChE) (EC 3.1.1.7) plays an essential role in the central and peripheral nervous systems where it terminates neurotransmission by rapidly hydrolyzing acetylcholine (ACh) (Fig 1) [1]. AChE is widely investigated with over 60,000 publications on the subject. AChE presents itself as a compelling example of enzymatic catalysis (Fig 1) and as a therapeutic target to control neurodegenerative diseases [2]. In addition, agricultural pesticides that target insect AChE for inhibition also pose a health risk for workers and consumers [3, 4]. Moreover, organophosphate (OP) based nerve agents such as sarin, VX, and soman were developed specifically to inhibit human AChE [5]. These nerve agents continue to be a threat, as observed recently in Syria [6]. Permanent inhibition of AChE results in “runaway” neurotransmission leading to cognitive deficiencies, seizures, paralysis, and eventually death depending on the exposure and rapidity of treatment (Fig 2) [7].

Decades of research in medical treatments for permanent AChE inhibition have produced direct and symptomatic treatments of OP intoxication. Symptomatic treatments, such as atropine and benzodiazapene, target other proteins and block the secondary effects (diaphragm paralysis and seizures) caused by high ACh concentration due to AChE inhibition. Direct intervention attempts to reactivate the OP-adducted AChE through the use of strong nucleophiles, such as oximes. These compounds act to remove the OP from the enzyme active site, thereby restoring enzymatic function. There are currently only a few approved oxime-based reactivators in use throughout the world. Two common examples are 2-PAM and Hi-6; the former is the only approved reactivator in the US and has been fielded for decades. These oxime-based compounds have limitations that include poor penetration into the brain, and they themselves can reversibly inhibit AChE. Moreover, for structurally distinct nerve agents, such as soman, tabun and cyclosarin, efficacy of currently available oximes is still limited [4, 8]. Thus, the rational design of direct chemical agent medical countermeasures requires knowledge of the effects of the individual adducts on the structure and the dynamics of irreversibly inhibited AChE [5, 9].

**Fig 3:**
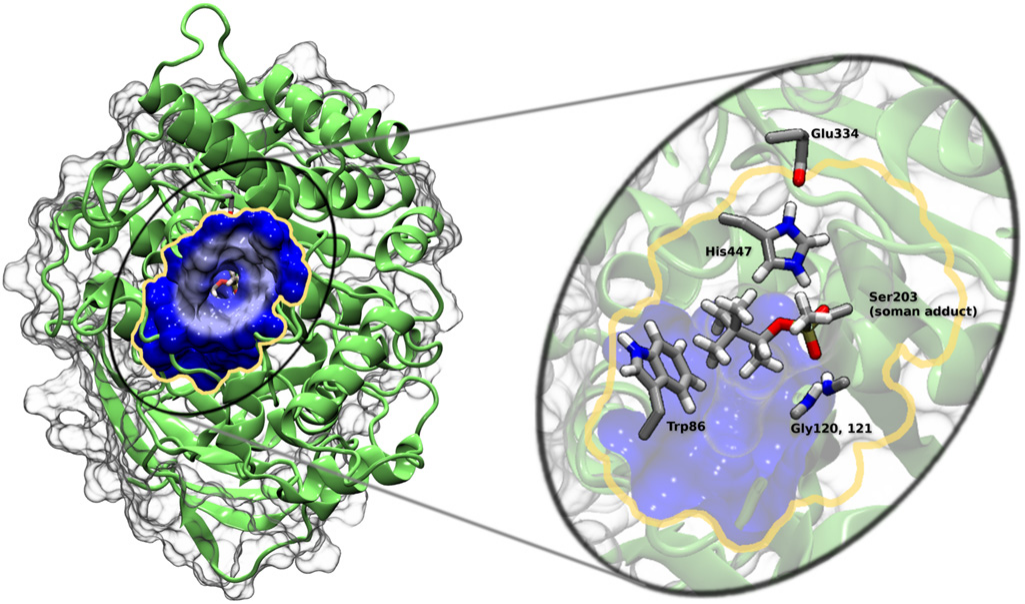
Human AChE structure showing the gorge entrance and important active site amino acid residues. The catalytic triad is composed of Glu334, His447 and Ser203. Soman is covalently bound to Ser203 with the pinacolyl tail interacting with Trp86 (choline binding site) and with the phosphate oxygen forming electrostatic interactions with Gly121 and Gly122 (oxyanionic hole). Figure created with VMD [11] and Tachyon [12].

The active site of AChE consists of a catalytic triad (Ser203, His447, Glu334, human sequence) at the bottom of the 20 Å deep gorge (Figs 1 and 2). An anionic “choline” binding site near Trp86, also at the bottom of the gorge, provides important contacts for the positively charged choline moiety of the substrate. The acyl binding pocket (Phe295, Phe297, and Trp236) stabilizes the acetyl group of ACh. Importantly, the oxyanionic hole (main chain nitrogen atoms of Gly121, Gly122, and Ala204) provides crucial stabilization of the acylation and deacylation transition states by stabilizing the buildup of negative charge on the carbonyl oxygen (Fig 3). These four sites are critical for enzyme catalysis, and their functions are easily rationalized based on the structural information gained from x-ray crystallography [10].

**Fig 4:**
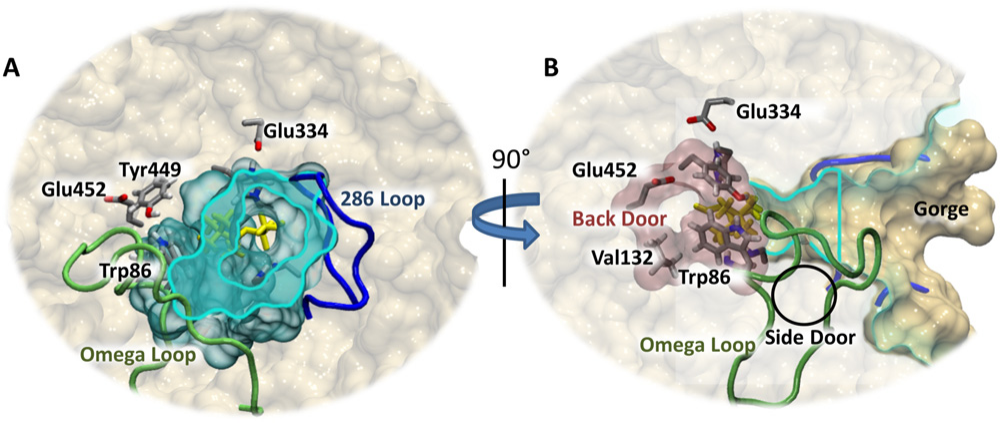
The surface of human AChE structure viewed from the (A) gorge entrance, (B) rotated 90° to the right, and (C) from the back door surface. The soman adduct is shown in spheres colored by atom name. The soman residue is at the bottom of the gorge (A), which is represented by a gray transparent surface. This gorge surface is cut at the cyan colored outline. This cut is also shown as a vertical line in part (B). Loop 286–298 (blue), Omega loop (green), and back door are proposed product exit paths in human AChE. The gorge is highlighted in cyan with the horizontal line in (B) showing the clipping plane in (A). The back door exit gate is composed of residues Trp86 and Tyr449 (shown as sticks). The opening to the back door, which has been described as a dimple on the surface of the protein [13] is defined by residues Val132, Tyr449, and Glu452 (shown as sticks). Figure created with VMD [11], Tachyon [12], and GIMP.

The catalytic rate of human AChE approaches the diffusion limit [14] (turnover number of 7.4 x 10^5^/min [15]). The protein must attract and quickly orient the substrate, break the required bonds, and remove the products from the active site. To attract acetylcholine, the whole protein is posited to create an electric field, which draws the positively charged substrate into the mouth of the active site gorge [16–19]. The peripheral anionic binding site (PAS) (Tyr72, Asp74, Tyr124, Glu285, Trp286, and Tyr341), located just inside the gorge entry, snares and properly orients the substrate [20]. The PAS is purported to allosterically affect the catalytic site [21]. The “aromatic patch” (Tyr133, and Tyr337) acts in conjunction with Tyr124 to regulate the flow of substrates into the catalytic site from the gorge [22, 23]. Tyr124 is also shown to be involved in the hydrolysis of ACh, carbamylation, phosphonylation, and oxime reactivation mechanisms [24–27]. The fast hydrolysis rate limits the information that can be obtained by x-ray crystallography experiments about the conformation and dynamics of the protein during catalysis. Inferred from the available data, AChE hydrolysis depends on conformational changes of the three-dimensional structure because sterically the substrate cannot reach the active site, which is located at the bottom of the 20 Å deep gorge (Fig 4). Conceptually, this deep gorge also makes the removal of products sterically challenging as the products are posited to travel through this gorge back out to the bulk without appreciably blocking incoming substrates. Crystal structures of AChE are available that show only portions of the original products in the active site, suggesting that cleavage products of the original substrate have already been removed [28]. In other experiments, the hydrolysis products are shown binding to other parts of the protein relatively near the active site [29]. These studies suggest the products leave the active site through exit paths absent in the vast majority of available crystal structures [30].

Organophosphates target the catalytic triad of AChE. The reaction between an OP and AChE leads to inhibition of the enzyme by forming a covalent adduct at Ser203. Further reacting of the adduct leads to irreversible inhibition. Recent work by Sirin *et al*. [26] describes the phosphonylation of Ser203 by soman. Using QM/MM calculations they observed an addition-elimination reaction mechanism that contains two energy barriers of approximately 6 and 9 kcal/mole. The second and larger energy barrier is the transition state for the leaving of the fluoride ion. They show a large movement of the Tyr124 sidechain that appears to stabilize the negatively charged fluoride ion. The QM region is relatively small with only Ser203, His447, Glu334, Tyr124, and soman treated quantum mechanically. Interestingly, no water molecules in the active site participate in the proposed reaction mechanism.

Sidechain rotations and lower frequency backbone fluctuations are important for the gating mechanisms observed in many enzymes and specifically for AChE ([31] and references therein). Previous computational studies show the importance of protein conformational changes in the catalytic mechanism of AChE [30, 32–36]. In AChE, an aperture and a swing gate is proposed for the gating mechanisms of the main gorge and alternative exits, respectively. Access to the catalytic active site from the gorge is regulated by an aperture consisting of a subset of 14 conserved aromatic residues, which create a bottleneck 10 Å from the surface of the protein [37] (Fig 4B). An MD simulation of the native AChE also shows the presence of this bottleneck and suggests that it is open only 2.4% of the time in a 750 ps trajectory [38]. Steered MD simulations of AChE with small molecules, huperzine and E2020, bound in separate simulations also suggest that the energy barrier for the aperture gating is relatively low [39–41]. MD simulations of tetramethyl amine (TMA) show that the barrier created by this bottleneck is about 4 kcal/mol, which is in agreement with the catalytic rate of the AChE [42], although only four of twelve simulations are productive in allowing the TMA to leave the active site through the gorge. Van Belle *et al*. also reported on multiple copy sampling MD simulations of various ligands in the active site [43]. They find that both TMA and acetate ion stay confined within the active and PAS sites. Other smaller molecules (methane and acetic acid) exit the gorge readily. One caveat is that these simulations do not include explicit solvent. In both reports, the small number of TMA molecules leaving the gorge may be due to the electric field, which can act against a positive charge transiting the gorge from the active site [44].

Previous computational studies in combination with x-ray crystallography data suggest that additional openings other than the main gorge may play a role in moving compounds into and out of the active site. Three general areas of the protein are implicated in the removal of cleavage products and are shown in Fig 4B. The first alternative is the “back door” (Val132, Tyr449, and Glu452) located approximately 180° from the gorge mouth and ~14 Å from Ser203 and is proposed as an exit for larger leaving groups [13, 23, 45](colored pink). A swing gate composed of Tyr449 and Trp86 lies between the active site and the back door exit [30, 46]. Early x-ray crystallography experiments posited the presence of this exit as a flap that comprises most of the Omega loop [10, 45]. However, kinetic experiments on AChE mutants that attempt to close this exit by restraining the Omega Loop with disulfide bonds show that there is little to no effect on AChE activity [47, 48]. These kinetics experiments appear to contradict early MD simulations of AChE by Gilson *et al*., where the authors observe a transient opening to the surface of the protein near Trp86 and show a strong field is present to attract the positively charged choline reaction product [44]. Interestingly, the back door exit is the preferred exit route in recent MD simulations performed by Xu *et al*. [46]. In the Xu *et al*. study, 40 independent MD simulations were performed with thiocholine in the catalytic site. The unrestrained trajectories show that in 29 of the simulations the thiolcholine exits the active site through the back door, and one trajectory shows the thiocholine molecule leaving through the gorge. Van Belle *et al*. also observed methylamine exiting the active site via the back door [43]. In addition to the back door, a “side door” located 15 Å from Ser203 and approximately 90° from the gorge entrance is composed of residues from the Omega loop (Cys69 to Cys96) and may also allow access to the protein surface from the active site via widening of the loop at its middle or by a folding of the top of the loop back onto itself [49, 50] (Fig 4B). Indeed, in the aforementioned study by Xu *et*. *al*, seven of the trajectories show the positively charged thiocholine molecule exiting through the side door [46]. However, these MD simulations did not include an acetylated Ser203, which would be present given the free thiocholine in the active site. A third candidate exit site is composed of the sidechains in the 286–298 loop found on the opposite side of the catalytic site from the Omega loop (Fig 4A) [50]. This exit is observed in MD simulations of methylamine [43]. The route of product removal may also be dependent on the cleavage products [48].

As these structural regions are relatively small, alternate conformations may not be easily observed due to low occupancies derived from the electron density. However, MD simulations are ideally suited for interrogating small molecule perturbations to protein structure and dynamics [51, 52, 53].

In this report we describe our use of QM/MM and unrestrained classical MD simulations to capture the highlights of the phosphonylation reaction mechanism and to compare and contrast protein structural motions in the apo and soman-adducted forms of human AChE. In a single MD simulation such motions can be masked by thermal fluctuations. Thus, we rely on multiple metrics and their distributions over an ensemble of 40 simulations in the apo state and 40 simulations in the soman-adducted state. We also have performed longer MD simulations of both the apo and soman-adducted human AChE to understand the time scales of various protein motions.

## Methods

### Soman adduction reaction barrier calculation by electrostatically embedded QM/MM calculations

The human structure of AChE (PDB ID 1B41) [54] was downloaded from pdb.org [55] and used in preparing the MD simulations of the apo and soman-adducted hAChE proteins. This crystal structure contains the fasciculin toxin, which was removed for the current simulations. Protonation states were set by consideration of the environment surrounding each ionizable residue; Glu202 was not protonated. The PsCs diastereomer of soman was modeled into the active site of the human AChE structure and subjected to molecular dynamics simulations as previously described [56]. An energy minimized structure from that previous study was used as the starting structure for the QM/MM work.

We divided the atomistic system into two parts: (1) a primary (QM) system encompassing the active site and several of its neighboring residues (613 atoms), and (2) a secondary subsystem (the remaining atoms) that were not explicitly included in the QM calculation. All residues within 9 Å of the hydroxyl oxygen in Ser203 were considered the active site of hAChE and included in the QM region (Met85, Trp86, Asn87, Trp117, Ile118, Tyr119, Gly120, Gly121, Gly122, Phe123, Tyr124, Tyr133, Asp134, Gly201, Glu202, Ser203, Ala204, Gly205, Ala206, Ala207, Ser208, Gln228, Ser229, Gly230, Ala231, Trp236, Ala237, Phe295, Arg296, Phe297, Ser298, Val330, Val331, Glu334, Tyr337, Phe338, Val407, Val408, His447, Gly448, Tyr449, Glu450, Ile451, Glu452). The PBE functional was used for the calculations of the QM region [57]. For the secondary subsystem, a list of atomic positions and associated partial charges was generated (using CHARMM27 parameters). Based on that list of point charges, we generated a charge distribution given by a linear combination of Gaussian charge distributions associated with each point charge. The total charge of this distribution is equal to the sum of the point charges and is the opposite of the total charge of the QM system, resulting in a neutral system. The sum of all the Gaussian charges was evaluated at each node of a uniform finite difference mesh that covered the physical domain. We used a mesh spacing of ~ 0.12 Å, which resulted in a global uniform mesh of size 704 x 576 x 672.

The Coulomb potential associated with that charge field was then calculated by solving a Poisson equation on this mesh by finite differences, using a multigrid solver. The resulting Coulomb potential was used as the external potential for the QM calculation. To prepare the QM system, we terminated the bonds cut in the process of partitioning the atomistic simulation into a primary and secondary subsystem as described previously [58]. Water molecules were either fully included in the QM region or not at all. Within our pseudopotential approximation and neglecting spin, we had 854 electronic wave functions to calculate. The QM domain was only a fraction of the original MM system, with dimension 38.68 x 30.94 x 30.94 Å^3^, which corresponds to a subset of the mesh points used for the Coulomb solver. The resulting mesh covering the QM computation domain is of size 320 x 256 x 264. Since the QM mesh is a subset of the global mesh, the external electrostatic field was defined on the QM calculation by a simple injection of the global Coulomb potential. Once this external potential was set, the geometry of the termini atoms was optimized in this Coulomb field with all the other atoms fixed. Finally, we locked the positions of a shell of atoms at the boundary of the QM region, and let only the atoms in the inner regions move freely, including the active site residues. This system resulted in a total of 222 free QM atoms. All QM calculations were carried out using the MGmol code, that implemented an O(N) complexity Density Functional Theory solver [59].

The reaction coordinate for the QM reaction path calculation was defined as the difference of the distance between the Ser203 hydroxyl oxygen atom and the phosphorous atom of soman and the distance between the phosphorous and fluorine atoms; d(Ser203-O,P1)—d(F2,P1). The geometry of the molecular structure, for a finite number of values of that reaction coordinate between reactants and products, was computed by geometry optimization with a constraint on each reaction coordinate.

These geometry optimizations determined the conformations of the soman adduct and other key residues (described above) in the active site for the two transition states and the conformation of the penta-coordinate intermediate after the departure of the fluoride ion. The final structure from the QM/MM geometry optimized reaction coordinate is the phosphonylated Ser203. This conformation of the soman-serine adduct in hAChE is in qualitative agreement with the previously published crystal structure of the non-aged soman-adducted *Torpedo californica* AChE (PDB ID 2WG2 [60]). The final structure from the QM/MM geometry optimization was used as the starting point for the adduct MD simulations after setting the pinacolyl tail to the PsCr diastereomer.

### Molecular dynamics simulation of apo and covalently bound soman

All classical molecular dynamics calculations in this study were performed using the program NAMD 2.7 [61] with the CHARMM27 force field [62]. The apo (PDB ID 1B41) and soman-adducted hAChE monomer (final structure from QM/MM calculations) was solvated in a TIP3P [63] water box (81.8 x 70.1 x 87.8 Å^3^) sufficient in size to have at least 12 Å of water between the protein and interface. Counter-ions were added to the solution to a 0.15 mM concentration. The total number of atoms was about 57000 for the two systems.

Parameters for the soman-adducted serine were derived from *ab initio* calculations (HF 6-31G*). Partial charges were derived from RESP calculations [64] with additional modifications for compatibility with the CHARMM27 force field. These parameters are comparable to previously published MD simulations of a sarin-adducted mouse AChE [65].

The apo and soman-adducted hAChE systems were equilibrated in several steps. First, the energy of the total system was minimized for 2000 steps. Using this minimized structure as a constrained reference for the hAChE heavy atoms (40 kcal/mol * Å ^2^), water molecules and ions were allowed to move in a 1 ns MD simulation. Constraints on the hAChE heavy atoms were reduced in five 1 ns steps. Finally, 80 production (40 apo and 40 soman-adducted) MD simulations were propagated for at least 48 ns with the following settings: 298 K temperature, 1 fs timestep, non-bonded cutoff starting at 10 Å and ending at 12 Å, pairlist set to 14 Å and updated every 20 cycles using the Langevin barostat and thermostat. Particle mesh-Ewald summation was used to treat the electrostatic potential energy and full updates occurred every 2 fs. Equilibrated PDB files for the apo and soman-adducted hAChE systems and other data are located at http://bbs.llnl.gov/data.html.

Protein secondary structure was calculated by the STRIDE [66] algorithm as implemented in VMD. This algorithm uses hydrogen bonding as well as dihedral angles in an energy function to compute secondary structures in proteins. Cross-sectional areas were calculated by measuring the area of triangles formed between three or more atoms and are in units of Å^2^. The areasperimeters of the triangles are summed together to generate the overall area. The average cross-sectional area values were calculated for each trajectory and averaged across all of the apo or soman adduct trajectories.

### MM/GBSA Calculations for the 80 MD simulations

Free energies were calculated using the Molecular Mechanics-Generalized Born/Solvent Accessible surface area (MM/GBSA) protocol as described by Habtemariam *et al*. [67]. The electrostatic contribution to the solvation free energy was calculated using the Generalized-Born molecular volume (GBMV) [68, 69] method within CHARMM (version 31b1) [70]. The non-polar contribution to the solvation free energy was calculated using the equation E_np_ = γSA, where γ = 7.2 cal/Å^2^, and SA is the solvent accessible surface area using a probe radius of 1.4 Å. Each MM/GBSA calculation used 500 snapshots from the non-adducted soman hAChE complex and the soman-adducted hAChE protein. The difference in the overall energetics was calculated by subtracting the average GBSA total energy of the non-adducted soman hAChE protein complex from the average GBSA total energy of the soman-adducted hAChE protein.

### Essential Dynamics of hAChE MD simulations

For the essential dynamics, we extended simulations for one apo and one soman-adducted protein system to 300 ns. We analyzed 100 ns intervals of each simulation because functionally important breathing motions of the protein can be analyzed more precisely with their corresponding amplitudes in these time scales. Specifically, in this study we analyzed the gorge volume fluctuations in the longer simulation for the apo structure and observed breathing-like behavior in the volume. The time required for completing this breathing motion was around 100 ns.

Covariance matrices were calculated for hAChE from the MD simulations and “diagonalized” using the ptraj utility [71]. The essential space is defined with only a few collective degrees of freedom of the Principal Component Analysis (PCA), which contribute most to the total atomic displacements seen in the trajectory. The eigenvectors of the covariance matrix are a de-convolution of atom fluctuations of the protein while amplitudes are proportional to the eigenvalues. The method is based on the notion that the largest fluctuations from the equilibrium structure of the protein represent the slowest, functionally important dynamical transitions of the protein [72, 73].

We analyzed an equilibrated 100 ns trajectory for each simulation to assemble the collective degrees of freedom for our analysis. To evaluate the convergence of the essential subspaces of the 100 ns long trajectory, we used the first 10 of the largest amplitude vectors of the covariance matrix. We divided each of the 100 ns long trajectories into two 50 ns sections. Then, to evaluate the overlap of these two segments, we calculated the root mean square inner products of the 10 vectors as:

$$RMSIP\;=\;{\left(\frac{1}{10}\sum_{i\;=\;1}^{10}\sum_{j\;=\;1}^{10}{{(\eta }_{i}\nu_{j})}^{2}\right)}^{\frac{1}{2}},$$

where η_*i*_ and ν_*j*_ are the eigenvectors of the two subparts.

Normalized correlation matrices of the system were also calculated by the eigenvectors of the covariance matrix of 100 ns long trajectory:

$$C_{ij}\;=\;\frac{\sum_{l\;=\;1}^{10}\frac{U_{il}U_{jl}}{\Omega_{ll}}}{({\sum_{m\;=\;1}^{10}\frac{U_{im}U_{jm}}{\Omega_{mm}})}^{\frac{1}{2}}{\left(\sum_{n\;=\;1}^{10}\frac{U_{in}U_{jn}}{\Omega_{nn}}\right)}^{\frac{1}{2}}},$$

where U is the matrix of eigenvectors, and Ω is the diagonal matrix of eigenvalues. The cross-correlations maps indicate correlation of motion between different parts of the protein.

We used these 10 largest amplitude eigenvectors as our essential subspace of our trajectory for the correlation analysis. Moreover, PCA was carried out with two different representations of the protein. In the first case, the whole protein was analyzed by using Cα atoms only (residues 5–541). In the second analysis, PCA was carried out with all heavy atoms within 20 Å of the Ser203 hydroxyl oxygen atom for the apo and soman-adducted hAChE. The second analysis can be seen as a focus on the active site of the protein with a more-detailed representation of the system.

## Results

### Soman hAChE adduction QM/MM calculations

**Fig 5:**
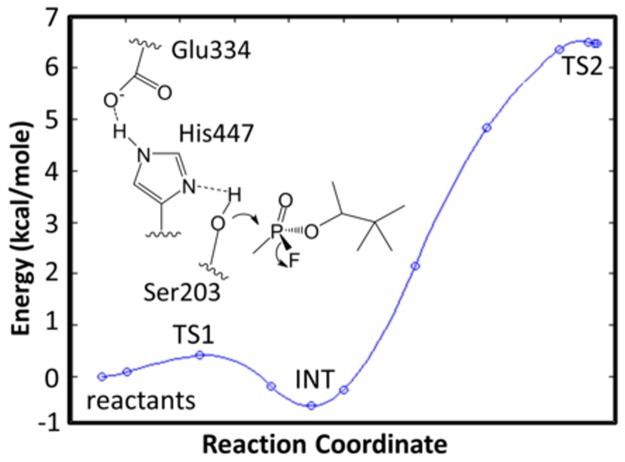
The energy barriers for the adduction of soman to the active site Ser203 by QM calculations. Each step is a single DFT geometry optimization calculation. The structure (S1 Fig) from the final geometry optimization was used as the starting point for the classical MD simulations of the soman-adducted human AChE. The 6.5 kcal/mole barrier value is similar to the experimentally determined value of 9.1 kcal/mole for that of sarin.

The reaction coordinate for the phosphonylation of hAChE by soman and leaving of the fluoride ion is determined using embedded electrostatic-QM/MM calculations. The energy barriers for the soman adduction of hAChE are 1 and 6.5 kcal/mole for the Ser203 nucleophilic attack on the phosphorous atom and the leaving of the fluoride ion, respectively (Fig 5). In Table 1, we show several key bond lengths from the QM/MM calculations. Here, we highlight several of interest. The Glu334-His447 hydrogen bond in the reactant structure is 1.60 Å and decreases to 1.54 Å in the intermediate and to 1.51 Å in the second transition state (TS2). The abstraction of the hydroxyl hydrogen atom from Ser203 by His447 results in a bond length of 1.47 Å in the reactants and intermediate structures and 1.56 Å in the TS2 structure. The fluoride to phosphorous distances are 1.63 Å, 1.79 Å, 2.62 Å, and 2.67 Å in the reactant, intermediate, TS2, and product states, respectively. We also observe that an active site water molecule interacting with the hydroxyl group of Tyr124 rotates and assists in the removal of the fluoride ion from the phosphate atom (S1 Fig). Without this water molecule, our calculations show that the energy required to remove the fluoride ion is greater than 20 kcal/mole. An additional water molecule also forms two hydrogen bonds between the sidechains of Glu202 and Tyr124. The final geometry optimized adduct structure is similar to the non-aged soman adduct of *Torpedo californica* AChE (PDBID:2WG2) [60] (See S1B Fig).

**Table 1:** Key bond lengths for atoms involved in the QM/MM calculations.

Bond	Reactants (Å)	Intermediate (Å)	TS2 (Å)	Products (Å)
**Glu334O-His447NH**	1.60	1.54	1.51	1.51
**His447N-Ser203OH**	1.47	1.09	1.05	1.05
**His447NH-Ser203O**	NA	1.47	1.56	1.56
**Ser203O-Phosphourous**	2.25	1.90	1.72	1.71
**Phosphorous-Pinacolyl O**	1.58	1.61	1.57	1.57
**Phosphorous-Fluoride**	1.63	1.79	2.62	2.67
**Fluoride-Water**	3.21	1.73	1.42	1.41
**Water-Tyr124OH**	1.94	3.03	2.51	2.47

### Structural and dynamical perturbations in apo and soman-adducted hAChE

**Fig 6:**
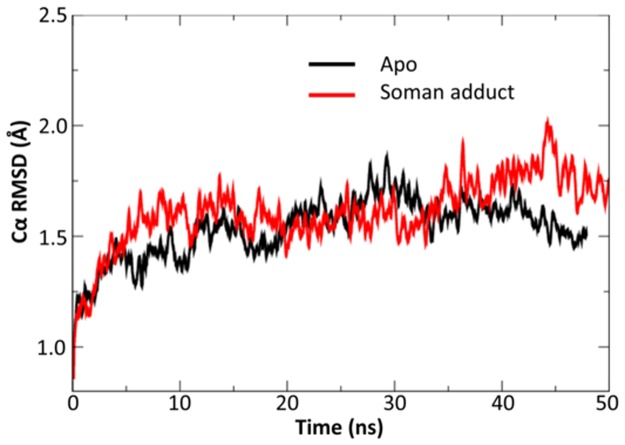
Example Cα rmsd data for the apo and soman-adducted hAChE MD simulations. After the initial adjustment, the longer-term values are stable. Notable low-frequency fluctuations can be seen in the Cα rmsd data for both simulations.

In general, the native and soman-adducted hAChE show remarkable protein backbone stability throughout the MD simulations. Three metrics are used to assess backbone motion: secondary structure analysis, root mean square deviation (rmsd) of the Cα atoms, and essential dynamics analysis. Secondary structure analysis shows little change in the total content of helical structure at 30–35% and beta structure at 1–3% in both the native and soman-adducted trajectories. The average Cα rmsd of all trajectories have values near 1.7 Å (Table 2). Interestingly, there are 0.5 Å fluctuations in the Cα rmsd values that do suggest longer time scale ~10–30 ns oscillations in the backbone dynamics for both the native and soman-adducted trajectories (Fig 6). Essential dynamics will be discussed in more detail below.

**Table 2:** Cα RMSD values and opening areas for the native and soman-adducted protein MD trajectories.^a^

Trajectory	Cα rmsd	Triad Cα rmsd	Omega Loop Cα rmsd	286 Loop Cα rmsd	Gorge Entry Cα rmsd	Back Door Cα rmsd	Gorge Entry Area	Back Door Area Cα	Back Door Area SC	Pinch Point Area	Side Door Area
**Apo hAChE**	1.73 (0.10)	1.50 (0.37)	1.78 (0.23)	0.83 (0.21)	1.80 (0.33)	1.1 (0.25)	182 (13)	21(2)	14 (4)	77 (7)	66 (7)
**TcAChE** ^b^	1.80	1.17	1.46	0.86	1.39	1.21	207	17	25	73	50
**Soman adducted hAChE**	1.70 (0.17)	1.16 (0.20)	1.28 (0.30)	0.53 (0.20)	1.52 (0.34)	0.87 (0.2)	221 (17)	19 (2)	24 (4)	74 (6)	59 (5)
**TcAChE** ^b^	0.90	0.30	0.80	1.46	1.16	0.43	209	17	24	64	50

^a^ The values exclude data points from the first 1 ns of each simulation and used every 10^th^ frame for a total of 4700 data points per trajectory. 40 trajectories for each protein state were performed for a total of 188000 data points. The root mean square deviation (rmsd) values are in units of Å and the standard deviations are shown in parentheses.

^b^ Rmsd values listed for the TcAChE proteins are calculated between the TcAChE crystal structures and the initial hAChE structure before MD is begun.

Triad contains Cα atoms from residues Trp86, Ser203, His447, and Glu334 (TcAChE:Trp84, Ser200, His440, and Glu327). Omega loop contains Cα atoms from residues 69 to 96 (Torpedo: 67 to 94).

286 loop contains residues 286 to 291

Gorge Entry contains Cα atoms from residues Tyr72, Leu76, His287, Glu292, and Tyr341 (TcAChE: Tyr70, Gln74, Asn280, Asp285, and Tyr334).

Side Door Entry contains backbone atoms from residues Asp74N, Leu76Cα, Met85Cα, and Asn87Cα (TcAChE: Asp72N, Gln74Cα, Met83Cα, and Asn85Cα)

Back Door contains Cα and sidechain atoms from residues Met85Cα, Val132Cg1, Tyr449Oh, and Glu452Cd (TcAChE: Val129Cg1, Tyr442Oh, and Glu445Cd).

Pinch Point contains backbone atoms from residues Trp86Hα, Tyr124Cα, and His447Cα (TcAChE: Trp84Hα, Tyr121Cα, and His440Cα).

The overall stability of the adducted protein was investigated by performing MM/GBSA calculations on the apo, soman-hAChE encounter complex, and covalently-adducted hAChE. We find the soman-adducted protein is more thermodynamically stable by ~-90 kcal/mole with an rms of ~33 kcal/mole in comparison to the encounter complex, which is only stabilized by ~-9 kcal/mole [56].

### Apo vs soman-adducted hAChE backbone dynamics

**Fig 7:**
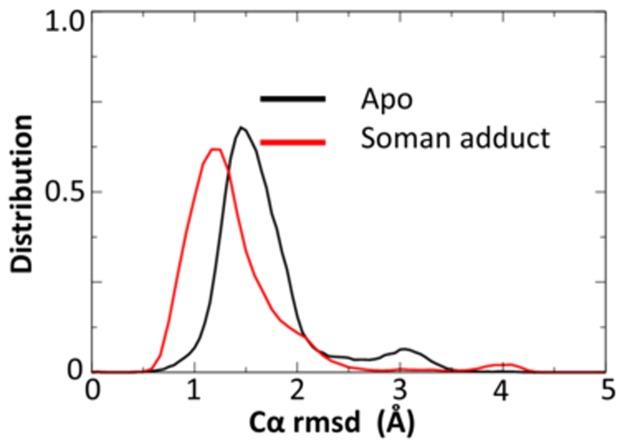
Comparison of motions of apo and soman-adducted hAChE. Cα rmsd values for the Omega loop are shown. In the soman-adducted protein the Omega loop motions are significantly lower than those in the apo protein. A second peak at 3 Å in the apo structures suggests an additional conformation is present.

A close inspection of the 50 ns trajectories in several structural regions reveals several significant differences between the apo and soman-adducted structures. The catalytic triad residues (Ser203, His447, Glu334, and Trp86), Omega loop (residues 69 to 96), gorge entry (residues Ty72 leu76 His287 Glu292 and Tyr341) and 286 loop (residues 286–291) in the soman-adducted structures have significantly lower Cα rmsd values compared to their respective residues in the apo trajectories (Table 2). We find that the distributions of the Cα rmsd values differ as well. For example, in Fig 7 we show the distribution of Cα rmsd values for the Omega loop residues with a large peak around 1.5 Å with a second small peak around 3 Å in the apo structures. However, the soman-adducted structures show the larger peak shifting to a lower Cα rmsd value, ~1 Å, while the second peak at the higher Cα rmsd diminishes. As the side door (residues Asp74, Leu76, Met85, and Asn87) is wholly defined by the Omega loop (Fig 4), its Cα rmsd values are correspondingly lower in the soman-adducted protein than the apo structures. Moreover, the Cα rmsd values of the back door residues (Met85, Val132, Try449, and Glu452) are slightly larger in the apo than the soman-adducted hAChE structures (Table 2).

We use the apo and soman-adducted crystal structures of *Torpedo Californica* (TcAChE) (PDB ID 1EA5 and 2WG2) [60, 74] in our analysis as a comparison with our respective MD structures of the human AChE. In Table 2 we compare the crystal structure of TcAChE to the hAChE initial starting structures used in the MD simulations. Generally, the Cα rmsd values show reasonable deviations from torpedo structures. However, there is a difference in the whole protein Cα rmsd between the apo TcAChE crystal structure and initial apo hAChE structure of 1.8 Å.

### Apo and soman-adducted hAChE entrance surface areas

We measure the average cross-sectional areas of the various entrances to the active site of the protein as shown in Table 2 and illustrated in S2 Fig. The most striking differences are found in the calculations of the gorge entrance and back door sidechain areas. The gorge entrance is larger in the soman-adducted structures in comparison to the apo structures, 221 Å^2^ vs 182 Å^2^. Even though the average back door area using Cα atoms is similar in the soman-adducted hAChE and apo structures, 19 Å^2^ vs 21 Å^2^, the average cross-sectional areas of the sidechains are significantly larger in the soman-adducted hAChE structures, 24 Å^2^, compared to the apo structures, 14 Å^2^. Also, the average pinch point areas at the bottom of the gorge have similar values in both sets of simulations at 74 Å^2^ and 77 Å^2^, soman-adducted and apo hAChE, respectively.

**Fig 8:**
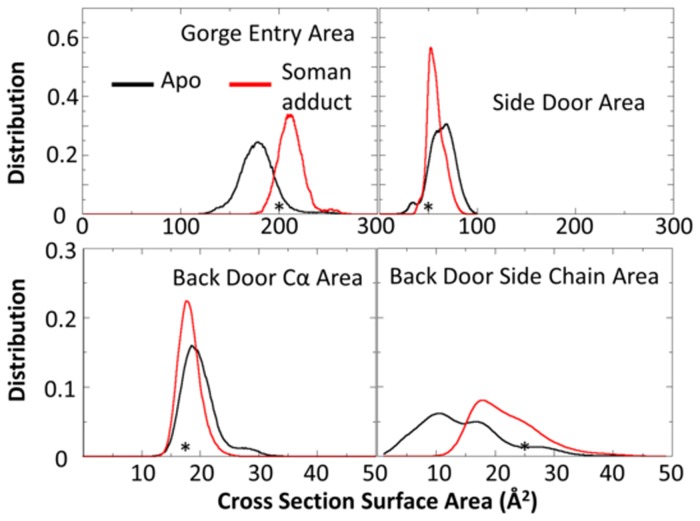
Cross-sectional area distributions for three protein regions are shown for the apo (black) and soman-adducted (red) MD structures. Only the gorge entry and back door sidechain regions of the soman-adducted hAChE protein experience a shift to larger cross-sectional area values relative to the apo trajectories while the other areas are similar or diminished in area. These data suggest the soman adduct is shifting the protein into a more restrained set of conformations. The asterisk denotes the cross-sectional area of comparable atoms in the TcAChE apo and soman adducted structures.

The distributions of the cross-sectional areas for the gorge entry, side door, and back door are informative and are shown in Fig 8. The gorge entry and back door sidechain cross-sectional areas in the soman-adducted structures shift to significantly larger values in comparison to the apo structures (Table 2 and Fig 8). In addition, the side door area is significantly altered so that a narrow range of values centered around 50 Å^2^ are sampled in the soman-adducted structures, while the apo structures sample a wider range of areas from 25 to 100 Å^2^. Furthermore, two separate peaks are present in the distributions of the side door cross-sectional areas in the apo structures, which may represent a sampling of closed and opened states (Fig 8). The sidechains of the residues that comprise the side door are small or form hydrogen bonds with adjacent residues in both apo and adducted structures. So, when the side door fluctuates, the sidechains move with the backbone and do not fill in the resulting opening.

In the back door region of the protein, we observe shifts in the cross-sectional areas in both the backbone atoms and in the sidechains of Glu452, Val132, and Tyr449 in the soman-adducted structures (see Figs 2, 8, and S2 Fig). The back door Cα area distributions show a shift towards smaller cross-sectional areas in the soman-adducted hAChE structures in comparison to the apo structures. In contrast to the Cα area measurements, the sidechains for the back door residues show an increase in cross-sectional areas in the soman-adducted structures, as measured from the ends of each sidechain. These observations are similar to that of the gorge mouth and the pinch point in that the outer surface has expanded in area while the internal structures have reduced cross-sectional areas. Together these data suggest that the majority of the soman-adducted hAChE protein is in a state with the gorge entry and back door sidechains are in a more open conformation in comparison to the apo structures. In contrast to the back door sidechains, the sidechains of the side door are small or form hydrogen bonds with adjacent residues in both apo and adducted structures. So, when the side door fluctuates, the sidechains move with the backbone and do not fill in the resulting opening.

#### Comparison of cross-sectional areas to Torpedo Californica crystal structures

The cross-sectional area values for the apo TcAChE are 207 Å^2^, 17 Å^2^, 25 Å^2^, 73 Å^2^, and 50 Å^2^, respectively for the gorge entry, back door Cα, back door sidechain, pinch point, and side door (Table 2). The corresponding values for the soman-adducted torpedo structure are 209 Å^2^, 17 Å^2^, 24 Å^2^, 64 Å^2^, and 50 Å^2^. Generally, the soman-adducted and apo TcAChE structures do not display much difference in these openings. However, these values are encompassed in the distributions from the MD simulations of the hAChE. We plot these values in the cross-sectional area distributions in Fig 8. The TcACHe gorge entry area rests in the intersection of the two hAChE distributions. The majority of the soman-adducted hAChE structure sample larger area values than those of the apo and adducted TcAChE structures. In the case of the side door and back door cross-sectional areas, the TcAChE values are more similar to the soman-adducted hAChE structures.

### Important Trp86 and Tyr449 interactions in apo and soman-adducted hAChE

**Fig 9:**
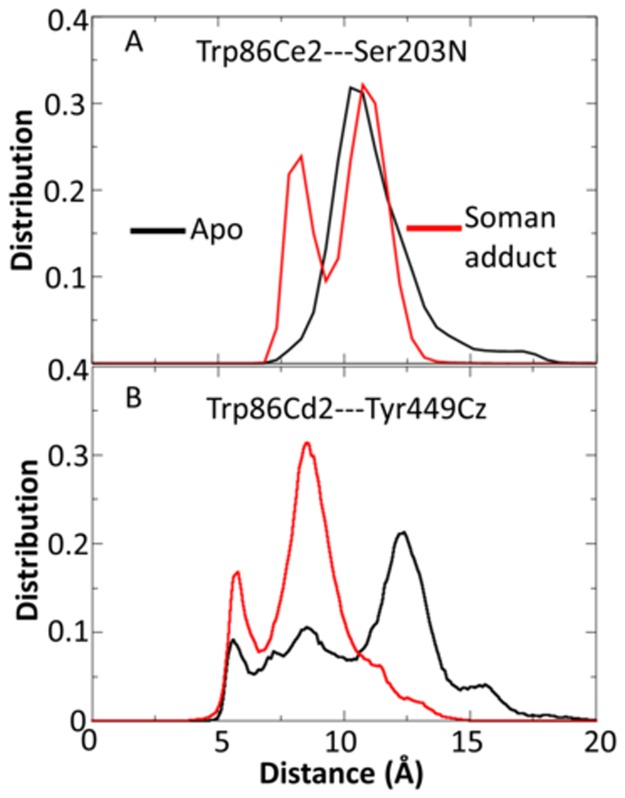
Sidechain to backbone and sidechain to sidechain distance distributions for Trp86, Se203, and Tyr449. (A) Distance distributions for the Trp86 sidechain to the Ser203 backbone. (B) Trp86-Tyr449 sidechain distance distributions in the apo and soman-adducted hAChE structures. The distances in the soman-adducted hAChE simulations show an increase in the probability of distances of 8 Å and of 5 Å compared to the apo distribution which is due to the Trp86 interaction with the soman pinacolyl tail. The separate peaks in the apo distribution are evidence for four different states in the apo hAChE structures.

Trp86 and Tyr449 are both involved in several important interactions near the active site and with the soman adduct. The distance between the Trp86 sidechain and the main chain of the catalytic site residue Ser203 can determine the extent of water access between the Trp86 sidechain and the pinacolyl tail of the soman adduct. Shown in Fig 9A, the Trp86 sidechain of apo structures samples a single broad distance distribution with a single peak centered near 10 Å. However, the presence of the soman adduct creates a second conformation for the Trp86 sidechain, creating a bimodal distribution with peaks at ~8 and ~11 Å^2^. The Trp86 and Tyr449 distance distributions are shown in Fig 9B because of their possible role as gatekeepers for the back door. The soman-adducted simulations have two peaks, one near 5 Å and the other near 8 Å, while the apo structures have four distinct peaks near 5 Å, 9 Å, 12 Å, and 16 Å. Namely, the apo structures are able to sample more conformations for the back door while soman-adducted structures are more restricted in back door conformations. Indeed, the loss of flexibility in the soman-adducted simulation is apparent from our correlation analysis which is presented below. In addition to its interactions with Trp86, the back door gate residue, Tyr449, is near a member of the catalytic triad, His447. In our simulations of the adducted hAChE structure, the sidechain of His447 forms hydrogen bonds to either Glu202 or the hydroxyl oxygen atom of the original serine sidechain that is presently bonded to the soman phosphorous atom. In the apo structures, these interactions fluctuate, which allows this loop and Tyr449 to have increased conformational sampling.

### Gorge dynamics in the apo and soman-adducted hAChE

**Fig 10:**
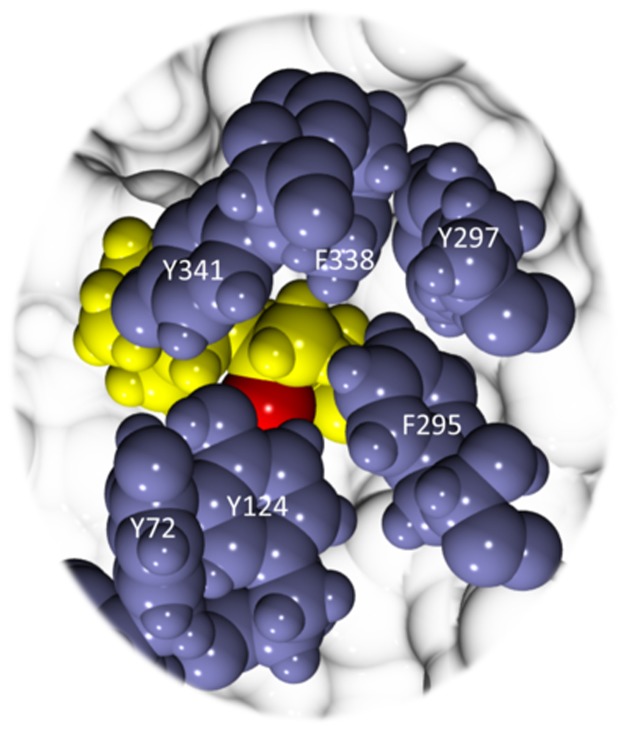
The soman adduct in hAChE as viewed from the gorge entrance. Soman (yellow space-filled spheres) is mostly occluded by aromatic residues (blue space-filled spheres). The phosphoryl oxygen of the soman adduct is colored red. The aromatic sidechains sequester the soman adduct from the gorge thereby limiting access of traditional countermeasures. Figure created with VMD [11] and Tachyon [12].

**Fig 11:**
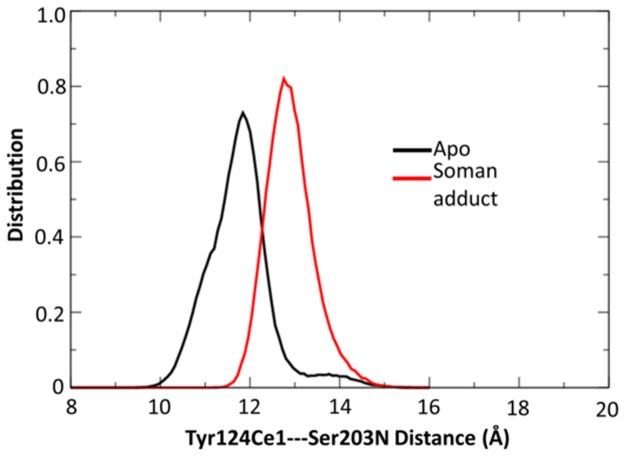
Distance distributions for the Tyr12Ce1 to Ser203N atoms. The soman-adducted structures show a significant increase in distances in comparison to the apo structures. This increase is due to the interactions of the phenol sidechain with the pinacolyl tail. A small peak at 14 Å in the apo distribution suggests a second conformation that allows ligand access to the catalytic triad.

Access to the soman adduct through the gorge is often blocked at the aromatic patch. We illustrate these interactions in Fig 10, which shows residues Tyr72, Tyr124, Phe295, and Tyr341 blocking direct access to the soman adduct from the gorge even though the gorge entry is more open than in the apo structures (Table 2). The distance distributions of the Tyr124 sidechain to the Ser203 backbone are shown in Fig 11 for both soman-adducted and apo structures. Residues Tyr124 and Ser203 in the soman-adducted proteins tend to be 11.5 Å to 16 Å apart with a single distribution peak centered at 13 Å. The larger average distance is due to the interaction of the phenol sidechain with the pinacolyl tail of soman, which prevents closer phenol sidechain distances to the Ser203 backbone of the soman adduct. Dissimilarly, in the apo structures, the distance between Tyr124 and Ser203 is between 9 Å and 16 Å with two significant peaks, one centered near 12 Å and a second smaller peak at 14 Å. In addition, distances are measured between the Cα atoms of key aromatic residues in the gorge (Tyr124, Phe338, and Tyr341) and no significant differences (all were ~15 Å) are seen between the apo and soman-adducted hAChE. We also measure the distances between the Tyr124OH and Phe338CE2 atoms as a probe for gorge radius (S3 Fig). For both structures we observe non-Gaussian distance distributions. The soman adducted trajectories show a peak at 7 Å and a small shoulder at 10 Å, indicating a larger gorge radius at this point due to the pinacolyl sidechain of soman, while in the apo structures there is a shoulder at 5 Å and also a peak at 7 Å.

### Sidechain conformational analysis of key aromatic residues

**Fig 12:**
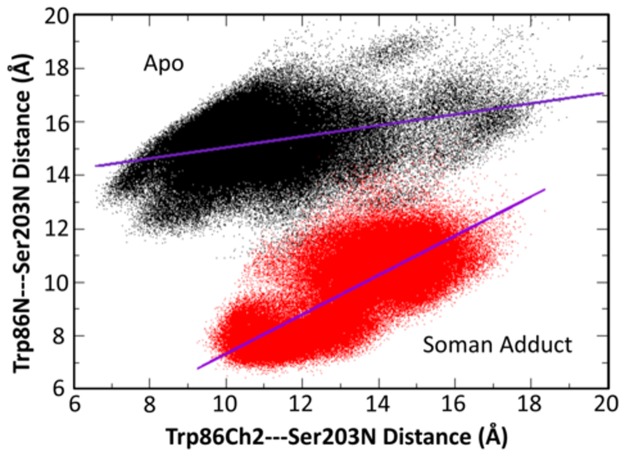
Correlation of the Trp86 main chain (N) and sidechain (CH2) atom distances to Ser203 (N). The high correlation suggests that the soman adduct has attenuated the sidechain motion of Trp86 and therefore directly influences the structure and dynamics of the Omega Loop and the swing gate to the back door exit.

The χ_1_ and χ_2_ angles for 14 conserved aromatic residues (Tyr72, Trp86, Phe123, Tyr124, Tyr133, Trp236, Trp286, Phe295, Phe297, Phe337, Phe338, Tyr341, Trp439, and Tyr449) in the apo and soman-adducted structures are shown in S4 Fig. These residues interact with the pincolyl tail of soman and generally display different sampling compared to the apo structures. In the adducted structures, Tyr72, Phe338, and Phe295 sample a broader χ angle space than in the apo structures, while Tyr124 and Tyr341 are more restrained. The analysis shows that Tyr449 is not very mobile in the soman-adducted structures relative to the apo structures. The sidechain conformations of Trp86 are affected by the soman adduct as we observe additional sampling of χ angles; χ_1_ 175–185°, χ_2_ 265–275°. This additional sampling is not present in the apo structures. The sidechain of Trp86 movement in relation to Ser203N is reduced, and as a whole Trp86 motion is more correlated to the backbone motions in the presence of the soman-adduct than in the apo structures (Fig 12).

### Examination of longer-timescale motions in apo and soman-adducted hAChE MD simulations

Using the root mean square inner product (RMSIP) approach explained in the methods section, each 100 ns trajectory is divided into two 50 ns sections and the root mean square inner products for the first 10 modes are calculated first. We find that these regions have a correlation of 0.63 and 0.66, suggesting the modes are sampling similar subspaces in the 100 ns intervals for the apo and soman-adducted states. The correlation between the apo and soman-adducted proteins for these 100 ns intervals is 0.39. We use these 10 largest amplitude eigenvectors as our essential subspace of our trajectory for the correlation analysis.

**Fig 13:**
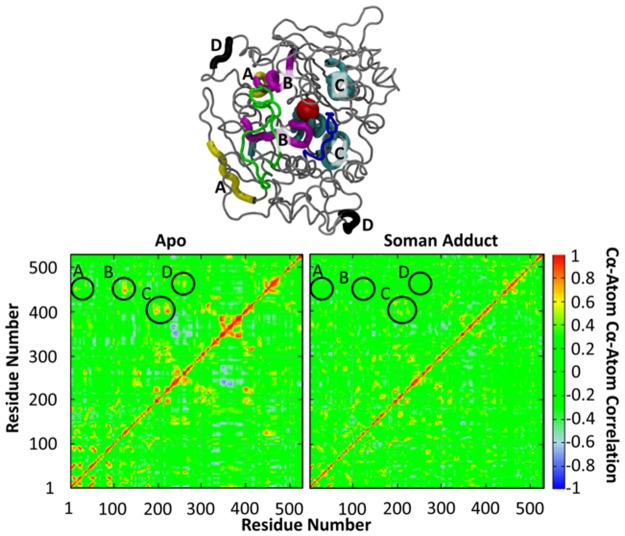
Normalized correlation matrices of the main chain Cα atoms in the apo and soman-adducted AChE. A backbone representation of the apo ache is shown (top) highlighting the circled regions (bottom); (A) residues 15–20 and 450–455, (B) residues 120–130 and 445–452, (C) residues 190–220 and 400–410, (D) residues 262–265 and 464–466.

A comparison of the correlation matrices of the Cα atoms in the apo and soman-adducted hAChE are shown in Fig 13. In general both correlated and anticorrelated motions are reduced in the soman-adducted structures, which is consistent with the reduced motion of the protein as shown in the rmsd analysis (Table 2). In addition, the correlated motions of specific regions of the protein, some of which are distant from the active site, are significantly altered in the soman-adducted hAChE. Several of these regions from the correlation plots (Fig 13) are highlighted as follows: (A) residues 15–20 and 450–455, (B) residues 120–130 and 445–452, (C) residues 190–220 and 400–410, (D) residues 262–265 and 464–466. We also color code the 3D regions of these residue groups in Fig 13 (top). Region A (blue) contains residues near His447 that are highly correlated with N-terminal residues opposite (back face) from the gorge entrance (front face). Region B (magenta) contains residues that define the gorge pinch point (Tyr124) and back door portions (449 and 452) of the protein both groups of residues contain or are near to members of the catalytic triad. Their high correlation in the apo protein supports the hypothesis that substrate and product trafficking can be coordinated via two different channels. Region C (green) comprises residues 190–200, which are on the same face of the protein as the gorge entrance (front) while residues 400–410 are on the opposite face (back). Region D (black) shows high correlation between two opposite sides of the protein that are each over 20 Å away from the active site. All of these regions are missing significant correlated motion in the soman-adducted protein. Moreover, a diffuse region constituting anti-correlative motions between residues 240–280 and 330–400 in the apo simulation are absent in the soman-adducted hAChE. These observations suggest that the soman-adduct has decoupled protein motions on each end of the protein that parallel the gorge entrance, gorge pinch point, and back door regions of the protein.

### Active site correlation analysis

**Fig 14:**
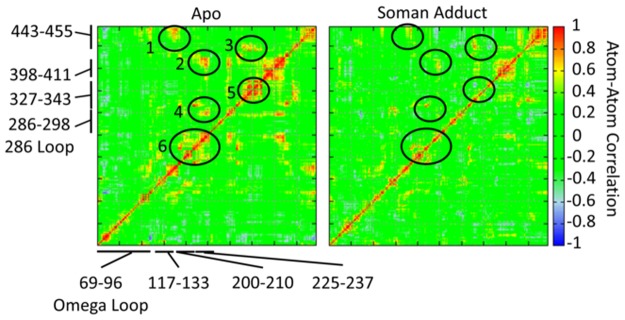
Normalized correlation matrices for all heavy atoms within 20 Å of the Ser203 hydroxyl oxygen atom for the apo and soman-adducted AChE. Residue numbers are shown for significant portions of the primary sequence. Positive correlations between near and distant residues is significantly decreased when soman is present. These areas of missing correlation are highlighted with ellipses. Additional negative correlation is observed between the 286 Loop and residues 443–455 in the soman adducted protein. The soman adduct appears to decouple positive correlative motions within a radius of 20 Å from the active site.

Correlation maps are shown in Fig 14 for all heavy atoms within 20 Å of the Ser203 hydroxyl oxygen atom for the apo and soman-adducted hAChE. Unlike the data shown in Fig 13, this PCA analysis is carried out with all heavy atoms including Cα atoms. Positive correlations between near and distant residues are significantly decreased when hAChE is adducted. Six regions of significant positive correlation in the apo structures are shown in Fig 14. All regions show attenuation in the adducted hAChE. Region 1 contains residues Gln228, Glu452, and Ile454 and links the active site Trp236 to the Glu452 which is part of the back door. In Region 2 a connection is observed between Trp236 and Val411. Region 3 contains several residues from the aromatic patch in the gorge, Tyr337, Phe338, Tyr341, with correlations to Val331 and Phe430. Region 4 shows a correlation between Trp236, Phe295, and Phe297 in the acyl binding pocket. Region 5 contains Tyr337, Phe338, and Tyr341 that have high correlation to Glu334 in the catalytic triad. Region 6 contains Trp236 and its interactions with Glu228 and Ser229. Each of these regions, with the exception of Region 1 have residues with sidechains in contact with the soman adduct. Additional anticorrelated motion, moving in opposite directions, is observed between the 286 Loop and residues 443–455 in the soman-adducted protein. Overall, the soman adduct appears to predominantly decouple positive correlative motions within a radius of 20 Å from the active site.

**Fig 15:**
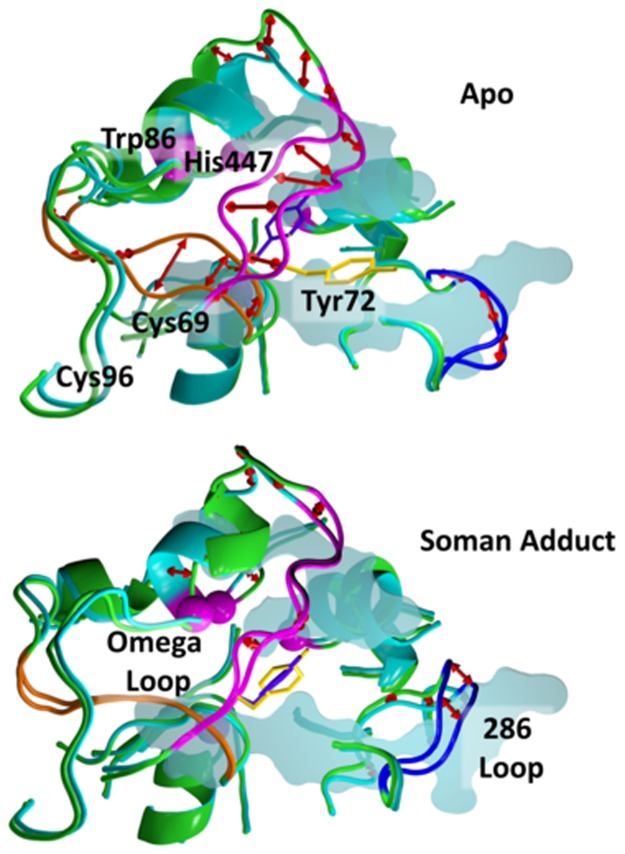
Displacement plots for the lowest-frequency mode of a 100 ns interval for the apo AChE (top) and Somand-adducted (bottom) structures. The covariance matrices were calculated for all heavy atoms within 20 Å of Ser203, including all the Omega and 286–298 loop heavy atoms. The protein backbone (cartoon) for the two end points of the mode are colored in cyan and green, except where large displacements are present in the apo AChE. In the apo structure, significant displacements are found in residues 71–77 are colored magenta and Tyr72 is shown in yellow sticks, loop 286–296 in blue, and residues 124–129 in orange. The Cα atom positions of the catalytic triad residues and Trp86 are shown as magenta spheres. A cross-section of the gorge is shown as a transparent surface slice. Displacement vectors are shown by red arrows. Major displacements are observed on the loops at the gorge entrance and back door regions of the apo protein. Tyr72 in the soman adducted AChE is shown and rotated to show the displacement of this side chain. The displacements in the soman-adducted structure are minimal compared to the apo structure suggesting stabilization of this region of the protein by the soman adduct in the lowest–frequency mode.

We also visualize the largest amplitude motion (first eigenvector of the covariance matrix) of the binding site area. As this eigenvector is well separated from the rest of the eigenvectors in terms of the amplitude of the motion, we assume that this is the dominant collective motion of the protein, which may play an essential role in its functionality. This mode in the apo structure shows the gorge mouth, gorge body, and catalytic active site are involved in coupled motions that can control substrate access to the catalytic triad (Fig 15). Major backbone motions are comprised of residues in the Omega loop (71–82), residues 124–129, and residues 289–292. Specifically, residues 77–82 at the apex of the Omega loop move towards the active site while residues 71–77 move away from the active site, extending this portion of the gorge mouth towards the solvent and closer to the 286 loop. These two motions in the Omega loop open and close the side door. Simultaneously, the 289–296 loop moves from the solvent towards the active site, and the approaching N-terminal side of the Omega loop constricts the mouth of the gorge. The sidechain motion of Tyr72 contributes to the decreased area of the gorge mouth. In concert with the loop motions, residues 124–129 approach the active site. The sidechain of Tyr124, which has shown to be important in substrate gating mechanisms [22, 23], obstructs access to the active site from the body of the gorge as this region moves. No significant motions are seen in the region encompassing the back door residues.

The motions found in the first mode in the soman-adducted hAChE (Fig 15 bottom) are very different than those observed in the apo hAChE (Fig 15 top). Displacement magnitudes in the apex of the Omega loop are significantly reduced. Also in contrast to the motions in the apo simulation, soman-adducted hAChE residues 71–77 in the Omega loop show little translational backbone motion. Instead, these residues show changes in phi–psi angles, causing residues to fill in the side door (e.g. Val76 sidechain rotation) and slightly widen the gorge mouth (Asp74 sidechain rotation). Tyr72 and Tyr124, which had significant displacements in the apo structures, remain fixed. The largest motion is found in the region of residues Val340 and Tyr341 and is mostly due to the large displacement of the Tyr341 sidechain. In this first mode, the displacement of the Tyr341 sidechain oscillates to open access to the active site near the apex of the Omega loop and contributes to the access restriction in the main gorge by interacting with Tyr124 and Tyr72.

## Discussion

### QM/MM calculations of soman phosphonylation of human hAChE

The results from the QM/MM calculations support an addition-elimination mechanism for the soman phosphonylation of Ser203 in hAChE (Fig 5). Qualitatively, our results are similar to a recent report by Sirin *et al*. [26] that also describes modeling the soman adduction of Ser203 in hAChE by soman. We both observe two energy barriers that are consistent with an addition-elimination reaction mechanism, and we both show that the second transition state where the fluoride leaves is the rate-limiting step (Fig 5). However, our calculations differ with Sirin *et al*. on four major points. Firstly, Sirin *et al*. do not include water molecules in the QM region. There are several water molecules in the 2WG2 soman-adducted TcAChE crystal structure that could play an essential role in the reaction. We include these water molecules in our simulations and find one critical water molecule stabilizes the reaction products. In general, water molecule positions as denoted by oxygen atoms in the active site of our QM/MM simulation are similar to that of the soman-adducted TcAChE structure. Water molecules not directly involved in the solvation of the fluoride ion around Tyr124 are conserved in our system and the crystal structure. Our QM/MM system does differ from the soman-adducted TcAChE structure in that there are no water molecules hydrogen bonding with the Glu202 sidechain carbonyl oxygen atoms. In the QM/MM structure the Glu202 sidechain forms hydrogen bonds with Tyr133 hydroxyl group and the amide group of Gly120. The exclusion of water molecules in the Sirin *et al*. QM region also influences the second, third, and fourth differences between our studies. Secondly, Sirin *et al*. observe a large movement of the Tyr124 sidechain in their study. Specifically, the Tyr124 phenolic sidechain shifts, and the hydroxyl group rotates in order to accommodate the large negative charge on the fluoride ion. In our calculations, only the hydroxyl group of Tyr124 needs to rotate in response to the fluoride leaving because the critical bridging water molecule sits between the fluoride ion and Tyr124 (S1 Fig). Thirdly, the rate-limiting energy barrier in our calculations (6.5 kcal/mol) is significantly lower than what Sirin *et al*. report (9.5 kcal/mol). Experimental values for the removal of choline for the natural substrate is ~12 kcal/mol [75], and sarin inhibition of AChE suggest a barrier value of 9.1 kcal/mole [76]. A lower rate-limiting energy barrier for soman adduction is consistent with soman out-competing ACh and having greater toxicity than sarin. Without the critical bridging water molecule, our calculations show that the energy required to remove the fluoride ion is greater than 20 kcal/mole, suggesting. This suggests that the lack of a water molecule in the Sirin *et al*. study may explain the higher transition state energy. Computationally, a lower rate-limiting energy barrier can be also due to the DFT calculation itself, which is known to underestimate transition state energies in general [77]. Sirin *et al*. use a Gaussian DFT method with the B3LYP functional and 6-31G* basis set which means that their value for the rate-limiting energy barrier is also underestimated at 9.5 kcal/mole. Therefore, the actual energy may be a value between 6.5 and 9.1 kcal/mole when the appropriate water molecules are included in the calculations. Finally, we see several structural differences in key bond lengths in the TS2 that may also contribute to the dissimilarity in transition state energies. Namely, the distance of 1.56 Å for the His447H-Ser203O bond is shorter compared to 2.2 Å in the Sirin *et al*. study. The His447H-pinacolylO bond distance is longer at 2.7 Å in comparison to 2.2 Å reported by Sirin *et al*. Also, the fluoride-phosphorous bond is slightly longer in our calculations at 2.6 Å, which is due to the interaction with the active site water. Again, this water molecule and others, which were not included in the Sirin *et al*. study, seem to be important in the mechanism and determining the TS2 energy. The lack of water in their calculations is also inconsistent with their previous work on the ageing mechanisms of soman adducted hAChE [78]. Moreover, our study includes a much larger QM region and residues that are one shell outside the residues immediately involved in the reaction, allowing a more precise calculation of the energy barriers (S1 Fig).

### Classical unrestrained MD simulations of apo and soman-adducted hAChE

The overall picture observed from the 40 apo hAChE and 40 soman-adducted hAChE simulations shows the adducted hAChE to be structurally stabilized and dynamically damped in the middle of the normal catalytic cycle. As the rate-limiting step of soman adduction is about half the energy of acetylation by ACh, this stabilization will occur quickly, given all other parameters are equal. The result is a protein that is caught waiting for Ser203 to be restored to a functional state by the removal of the soman adduct (see Fig 2). This point in the cycle is analogous to the removal of the acetyl group from Ser203 in the native acetylcholine hydrolysis reaction (see Fig 1). In this reaction the acetyl group is displaced by an activated water molecule, and the resulting acetic acid leaves the active site, either through the back or side doors (Fig 4B). As multiple acetylcholine molecules have been proposed to bind AChE simultaneously [20, 29, 79], a waiting acetylcholine molecule at the PAS in the gorge would then pass through the aromatic patch and pinch point (Fig 10) to the catalytic triad for hydrolysis by the regenerated Ser203. In the case of the soman-adducted serine, two possible reactions can occur in the presence of a water molecule in the active site: 1) the removal of the entire soman adduct via nucleophilic attack by an activated water or 2) the removal of the pinacolyl tail (i.e. ageing). The calculated energy barrier for the rate-limiting step for removing the entire soman adduct, by a Glu202 activated water molecule, is estimated to be 22 kcal/mole [80]. Three ageing mechanisms (protonation-deprotonation [81–83], push-pull [81, 84], and O-dealkylation [85]) have been suggested with the push-pull and O-dealkylation mechanisms requiring a water molecule. The rate-limiting energy barrier for ageing is estimated to be between 16 and 20 kcal/mole [78] suggesting that ageing is slightly more kinetically favorable. If the pinacolyl tail is removed first, the remaining phosphonate will be impossible to remove with current countermeasures, and the AChE is irreversibly inactivated (see Fig 2). Although our MD simulations do not allow for any part of the soman adduct to be removed, our data suggest that the soman-adducted protein is in a state of waiting for the adduct to be removed from Ser203 by a nucleophile from the traditional gorge [65, 86–88], side door, or the back door (Fig 4B).

### Increased thermodynamic stability of the soman-adducted human hAChE

The soman-adducted hAChE is thermodynamically stabilized by the covalently-bound soman by ~90 kcal/mol (rms 30 kcal/mole) compared to the non-covalent encounter complex which is stabilized by only 9 kcal/mol. Trapp, *et al*. attempt to quantify a ligand-induced increase in stability during thermal denaturation [89]. In their studies Huperizine A, a reversible inhibitor, is used to look for a ligand-induced increase in the thermal melting point of hAChE. However, no significant enhancement in the melting temperature is observed, which is possibly due to the noncovalent binding of Huperizine A. In our work, the soman adduct is covalently bound to the protein, and therefore its effects on dynamics and structure of hAChE is more evident as it is not shuttling in and out of the active site. Neutron scattering experiments similar to those that measured the glass state transition of hAChE [90] may be able to provide more information about the stability of the soman-adducted hAChE.

### Correlated motion of distant regions of the protein are reduced in the soman-adducted simulations

Correlations in backbone motions, as measured at the Cα level, in the soman-adducted hAChE structures are reduced when compared against the apo hAChE structures (Fig 13). In contrast, there are allosteric sites that are significantly correlated in the apo simulation, such as Regions A, B, C and D in Fig 13. The high correlations between residues in the gorge entrance or inside the gorge with residues at the back door support the idea there could be a way for the protein to shuttle substrate and product in between these two sites. The loss of these correlated regions, when the soman is covalently bound in the active site, can be interpreted as soman restricting the protein motions in such a way that these correlated segments cannot communicate with each other to act as allosteric sites. As suggested from these correlation graphs, the communication in the protein is happening via the flow of flexibility in between distant sites of the protein. One such example for the flow of communication is the flexibility in the Omega loop backbone enabling the large movement of Tyr72 in the apo simulations (Fig 15). The reduction in the correlation of backbone motions in the soman-adducted simulations can be a result of decoupling, e.g. the backbone atoms are still moving with the same magnitude but are not moving in a correlated fashion with respect to other regions of the protein. Another explanation is the motions of atoms in regions of the protein have been significantly dampened. Given that the Cα rmsd is lower in the soman-adducted trajectories, especially near the soman adduct (Table 2), it is more likely that reduced or missing correlations are a result of damped motions.

When the sidechains are included in the correlation motion analysis (Figs 14 and 15), correlated motions between sidechains near or in contact with the soman adduct are also significantly reduced in magnitude in comparison to the apo structures. This disruption of proximal and distant protein motions by going from apo to soman-adducted structures helps to elucidate the important residues responsible for transferring dynamical motions from one part of the protein to the other. For example, the reductions in the correlations at the residues near the active site end of the gorge, oxyanionic hole, and the back door contribute collectively to the loss of flexibility of the protein in the soman-adducted state. Fig 15 shows the protein motions that comprise the lowest-frequency mode in a 100 ns portion of the apo simulation. The Omega loop displays a higher flexibility, which plays a direct role in the dynamics of the gorge entrance and the side door. These motions also significantly affect sidechain dynamics. Namely, Tyr72 seems to gain more flexibility via the increased movement of the Omega loop and is able to interfere with the active site gorge entrance (Fig 12A). Our results are consistent with Tyr124 being important in the gating mechanism [22, 23]. Previous studies also report a general connection between backbone motions and gorge dynamics [23, 42] and are consistent with our results. The soman-adducted structure shows little movement in the Omega loop and in the 121–125 β-strand, as shown in Fig 15. In other words, the Omega loop loses its flexibility due to the presence of the soman in the binding site. But, motion of the 286–298 loop explains the increase in the cross-sectional area of the gorge mouth (see Table 2 and Figs 8 and 15).

### Specific effects of the soman adduct on gorge residues in human hAChE

The soman adduct also modulates the conformations of the aromatic residues that define the gating aperture to the active site from the gorge. Distance and χ angle measurements are used to define the motions of the sidechains to compare against previous MD simulations of the apo AChE and to search for perturbations of these motions due to the soman adduct. For example, Tai *et al*. (2001) found that the distance between the Tyr124Oh and Phe338Ce2 atoms is predictive for measuring the gorge radius [23]. We applied this metric to both apo and soman-adducted hAChE trajectories and show the frequency of distances in S3 Fig. The soman-adducted structures have slightly larger distances than the apo structures, which is consistent with the presence of the large aliphatic pinacolyl tail. Perturbations of the χ angles of residues Tyr124 and Phe338 as seen in our soman-adducted simulations are also reasonable (S4 Fig). At least two conformations of the Phe338 sidechain are modeled from the electron density in recent structural studies of cyclosarin-adducted mouse AChE by Artursson *et al*. [86]. The MD and crystalstructure data show that the conformations of residues in the gating aperture are indeed modulated by adducts in general, and in the case of the soman adduct, access from the gorge entrance is hindered.

Previous reports suggest that Tyr124 is involved in a gating mechanism that allows the substrate to enter the active site [22, 23]. Analysis of the distance between the sidechain of Tyr124 and Ser203 backbone (see Fig 11) suggests a possible gating mechanism in the apo structures. Two peaks are observed with the larger peak being less populated at 14 Å and is consistent with other reports. Zhou, *et al*. show the aperture is closed a majority of the time [38]. The Tyr124-Ser203 distances in the soman-adducted trajectories are slightly larger due to the presence of the large pinacolyl tail. The most pronounced difference in the Tyr124-Ser203 distances between the apo and soman-adducted structures is the lack of a second peak for the soman-adducted structures, suggesting no significant gating occurs in the soman adducted-structures. Our results suggest a fluid, auxiliary hydrophobic core is formed around the soman adduct which abrogates gating to the active site but enlarges the gorge mouth (see Fig 15 bottom). At the same time, the side door cross-sectional area is significantly shifted to a narrow open state that may provide an alternative access point to the active site.

### Soman adduct effects on the back and side doors in human hAChE

Early x-ray crystal structures and residual activity in fasciculin inhibited AChE suggest that there might be other entrances and exits (back or side doors) available for reactant entry and product exit [91–93] (see Fig 4B). Faerman *et al*. created disulfide mutants of AChE to covalently restrain the Omega loop because the Omega loop was thought to act as a flap that allowed product to be released. However, these experiments did not significantly alter catalytic efficiency [48]. Even though both the back and side doors may not be affected by the locked disulfide gate, they still may be responsible for the diminished but residual activity of AChE when fasciculin is bound. Our MD structures of apo hAChE are consistent with previous MD simulations and show the back door is rarely open [23]. Only a narrow cleft is present, based on the backbone cross-sectional areas measured in our apo structures. This same exit is more constricted in the soman-adducted structures (Fig 8). In the limited sampling of MD simulations, it appears that soman adduction does not promote opening of the back door. Only the swing gate that precedes the back door exit, comprised of residues Tyr449 and Trp86, is predominately open in the soman-adducted structures, whereas the apo trajectories appear to sample several different states (see Fig 9). However, the side door is physically closer to the adduct and nearly twice the size of the back door, making it a more logical exit for the entire soman adduct or pinacolyl tail (see Table 2 and Fig 8). Major differences between the apo and adducted structures are found in the Omega loop (side door) and its interfaces with the active site, gorge entrance, and putative back door of the protein. In Fig 7, for example, the overall Cα rmsd in the Omega loop for the adducted structures is lower than in the apo structures. The presence of soman affects the motions of the loop because of interactions with Trp86 and Tyr72 (Figs 2 and 10) as they are on opposite sides of the Omega loop. The sidechain interactions of these two residues with the soman adduct are directly transduced to the backbone atoms. Indeed, distances between the sidechain and main chain atoms of Trp86 and Ser203N are highly correlated with each other in the soman-adducted structures and are not correlated in the apo structures (Fig 12). These interactions stabilize the loop in those regions, thus lowering the average Cα rmsd values for the soman-adducted structures. The presence of a minor second peak at ~3.1 Å in the apo structures suggests of a second conformation for the Omega loop, which is not present in the soman-adducted hAChE structures (Fig 7). The presence of this alternate conformation is supported by the essential dynamics calculations of the heavy atom motions in the active site region and the proposed motion is highlighted in Fig 15. Moreover, x-ray crystallographic studies have shown that the sidechains of Trp86 and Tyr449 have significant fluctuations with respect to the active site Ser203 [30] in the apo form. The side door and swing gate to the back door, which are in stable and open states in our soman-adducted structures, may still function as alternate pathways for products to leave the active site during normal fluctuations (see Table 2 and Fig 8).

### Comparison to apo and soman-adducted TcAChE crystal structures

Comparison of the TcAChE crystal structures to the MD structures of the hAChE protein show that our MD results are reasonable. Indeed, calculated values of the TcAChE crystal structures are observed within the distributions of the MD data shown in Fig 8. Interestingly, both apo and soman-adducted TcAChE values are generally more similar to the soman-adducted hAChE protein, suggesting that dampened and decoupled motions in the somand-adducted hAChE MD structures may not be artifacts. Protein crystal structures are inherently more rigid due to the conditions in which the diffraction data are collected [60, 74] and this rigidity may be thought of as surrogate of soman adduction in the hAChE protein. The data from this comparison of protein from two species also suggests that the dampening and decoupling effects of the soman adduct may not be restricted to AChE proteins of a single species.

## Conclusions

We find that soman-adducted hAChE is thermodynamically and conformationally stabilized, especially in the region surrounding the soman adduct. This stabilization occurs due to the interaction of the soman adduct within the oxyanionic hole, residues in the Omega loop, and through increased non-polar contacts with nearby aromatic residues normally responsible for moderating substrate access to the active site. Since the protein core in the soman-adducted hAChE is stabilized, motions that are important for normal catalysis and therapeutic reactivation are dampened and decoupled. These modifications include aromatic and hydrophobic interactions with the soman adduct at the pinch point, limiting access to the soman adduct from the main gorge even though the gorge entrance is larger. Moreover, there is shift to two distinct states in the opening of the swing gate to the back door exit, as well as a significant increase in the area between the back door sidechains. In addition, there is an equilibrium shift in the side door cross-sectional area of side door equilibrium is shifted from a closed state to a narrower open state distribution. Both the back and side doors may be viable exits, if the soman adduct could be removed. Thus, these findings present a special case to understand the effects of covalent modifications on the network of connected motions that are responsible for the dynamics of enzymes. Applying this knowledge of exits and motions provides alternative entry pathways to the soman adduct for medical countermeasures. In the case of soman and in the interest of a universal reactivator, a successful, rational structure-based design effort would be focused on countermeasure-protein interactions at the side and back doors rather than in the gorge. Future work will address the therapeutic viability of these alternative pathways for the reactivation of soman-adducted hAChE.

## Supporting Information

S1 Fig A) Final step in the QM/MM soman (cyan) adduction reaction showing the water molecule that forms a bridge between the fluoride ion and hydroxyl group of Tyr124. This water molecule is in the same position as the oxygen of residue 546 in the 2WG2 crystal structure. B) The view of (A) is zoomed in to show overlay of the product from QM/MM simulation (d1.75 Å) of soman (green, Ps, Cs) with the Crystal structure of adducted AChE (Torpedo Californica, non-aged) with soman (cyan, Ps,Cr) PDBID 2WG2 [60].
(TIFF)

S2 Fig Atoms used in calculating the Cα rmsd and cross-sectional areas for the gorge entrance (A), side door (B), and back door (C) are shown with yellow spheres for soman-adducted AChE. Each image is rotated by approximately 90° to the right starting from the gorge entrance. The Omega loop is shown in green, 286 loop in blue, and the back door in mauve. The soman-adducted Ser203 residue is shown in licorice and colored by atom type.
(TIFF)

S3 Fig Tyr124 and Phe338 Sidechain-sidechain distributions. Distributions of the Tyr124OH distance to Phe338CE2 atom are shown for the apo and soman-adducted AChE.
(TIFF)

S4 Fig Soman adduct data for the aromatic χ_1_ and χ_2_ angles. The red sphere marks the χ_1_ and χ_2_ values in the crystal structure of human AChE (PDBID 1B41).
(TIFF)
