# Supplementary figures and images for: A Wrench in the Works of Human Acetylcholinesterase: Soman Induced Conformational Changes Revealed by Molecular Dynamics Simulations
Source: PLoS One. 2015 Apr 13;10(4):e0121092. doi: 10.1371/journal.pone.0121092 (PMC4395452; doi:10.1371/journal.pone.0121092)

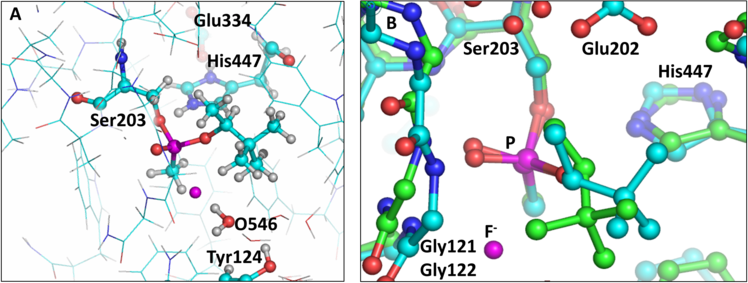

Supplement: S1 Fig — A) Final step in the QM/MM soman (cyan) adduction reaction showing the water molecule that forms a bridge between the fluoride ion and hydroxyl group of Tyr124. This water molecule is in the same position as the oxygen of residue 546 in the 2WG2 crystal structure. B) The view of (A) is zoomed in to show overlay of the product from QM/MM simulation (d1.75 Å) of soman (green, Ps, Cs) with the Crystal structure of adducted AChE (Torpedo Californica, non-aged) with soman (cyan, Ps,Cr) PDBID 2WG2 [60]. (TIFF) [file pone.0121092.s001.tiff]

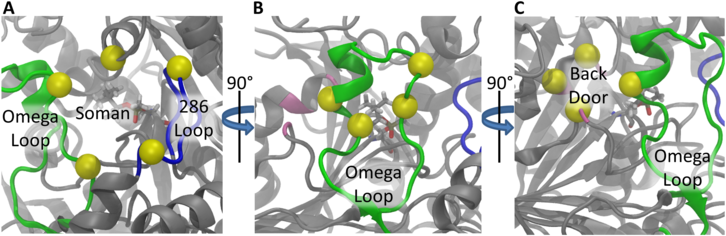

Supplement: S2 Fig — Each image is rotated by approximately 90° to the right starting from the gorge entrance. The Omega loop is shown in green, 286 loop in blue, and the back door in mauve. The soman-adducted Ser203 residue is shown in licorice and colored by atom type. (TIFF) [file pone.0121092.s002.tiff]

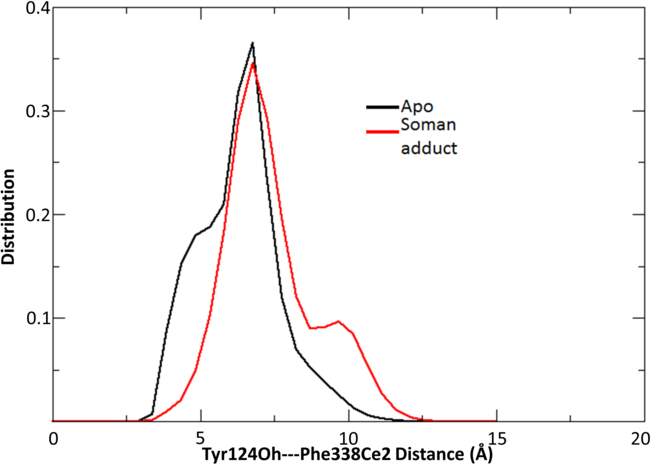

Supplement: S3 Fig — Distributions of the Tyr124OH distance to Phe338CE2 atom are shown for the apo and soman-adducted AChE. (TIFF) [file pone.0121092.s003.tiff]

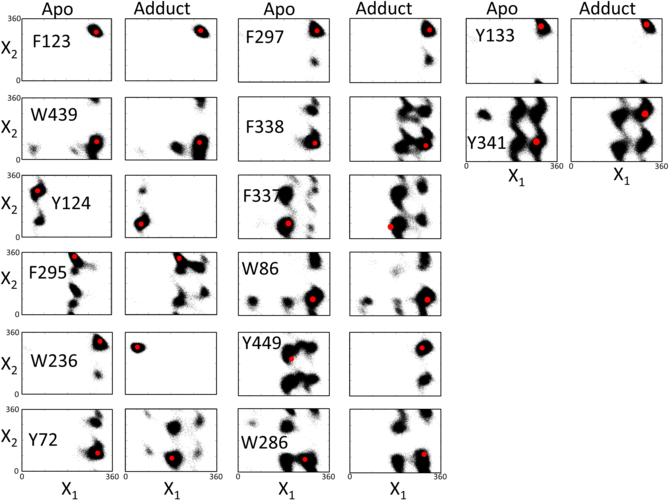

Supplement: S4 Fig — The red sphere marks the χ1 and χ2 values in the crystal structure of human AChE (PDBID 1B41). (TIFF) [file pone.0121092.s004.tiff]
